# Supplementary material for: Active site specificity profiling datasets of matrix metalloproteinases (MMPs) 1, 2, 3, 7, 8, 9, 12, 13 and 14
Source: Data Brief. 2016 Feb 22;7:299–310. doi: 10.1016/j.dib.2016.02.036 (PMC4777984; doi:10.1016/j.dib.2016.02.036)
Supplement: Supplementary file 10 — Supplementary material [file mmc10.zip › WebPICS_hMMP1_T_1%/P3prime.html]

 

PICS results


|  |  |
| --- | --- |
| **P3prime\_D**  31 in 277 sites   11.2 %    effects > 10 perc. pnts.   (vice-versa in brackets)  P2\_L: 22.1 (18.5)   P2prime\_L: 19.6 (13.8) |  |
| **P3prime\_E**  24 in 277 sites   8.7 %    effects > 10 perc. pnts.   (vice-versa in brackets)  P3\_A: 13.4 (10.1)   P2\_L: 19.9 (12.9) |  |
| **P3prime\_H**  9 in 277 sites   3.2 %    effects > 10 perc. pnts.   (vice-versa in brackets)  P1\_D: 26.8 (13.5)   P2prime\_Q: 27.9 (16.8) |  |
| **P3prime\_S**  23 in 277 sites   8.3 %    effects > 10 perc. pnts.   (vice-versa in brackets)  P3\_C: 17.4 (33.4)   P2prime\_T: 10.9 (13.9) |  |
| **P3prime\_T**  17 in 277 sites   6.1 %    effects > 10 perc. pnts.   (vice-versa in brackets)  P1prime\_C: 15.4 (43.9)   P2prime\_V: 41.1 (14.3) |  |
| **P3prime\_V**  22 in 277 sites   7.9 %    effects > 10 perc. pnts.   (vice-versa in brackets)  P2\_A: 19.0 (18.2)   P1\_G: 13.7 (12.1)   P1\_N: 16.1 (11.5) |  |
